# Supplementary material for: The Interruption of Transmission of Onchocerciasis in Abia, Anambra, Enugu, and Imo States, Nigeria: The Largest Global Onchocerciasis Stop-Treatment Decision to Date
Source: Pathogens. 2024 Aug 8;13(8):671. doi: 10.3390/pathogens13080671 (PMC11356909; doi:10.3390/pathogens13080671)
Supplement: Supplementary file 1 [file pathogens-13-00671-s001.zip › pathogens-3121635-supplementary.pdf]

Table S1. Onchocerciasis Ov16 ELISA epidemiological assessment results by local government area (LGA) for Abia, Anambra, Enugu, and Imo states, Nigeria, October 2020*

State	LGA	Villages/sites (n)	Dried blood spot samples (n)	Ov16 ELISA positive (n)	Ov16 ELISA positive (%)
Abia	Arochukwu	1	86	0	0
	Bende	1	110	0	0
	Ikwuano	2	233	0	0
	Isiala Ngwa North	2	342	0	0
	Isiala Ngwa South	3	291	0	0
	Isiukwuato	5	573	1	0.17
	Obi Ngwa	1	84	0	0
	Ohafia	3	327	0	0
	Osisioma	1	105	0	0
	Ukwa East	2	466	0	0
	Ukwa West	1	105	0	0
	Umu Nneochi	2	180	0	0
	Umuahia North	2	175	0	0
	Umuahia South	1	97	0	0
	TOTAL	27	3,174	1	0.03
Anambra	Aguata	1	100	0	0
	Anambra East	1	67	0	0
	Anambra West	3	235	0	0
	Anaocha	2	190	0	0
	Awka North	3	339	1	0.29
	Ayamelum	4	435	0	0
	Dunukofia	1	160	0	0
	Ekwusigo	1	130	0	0
	Idimili North	1	105	1	0.95
	Idimili South	1	143	0	0
	Ihiala	1	105	0	0
	Njikoka	1	130	0	0
	Nnewi North	1	105	0	0
	Nnewi South	1	79	0	0
	Ogbaru	1	110	0	0
	Onitsha North	1	105	0	0
	Onitsha South	1	105	0	0
	Orumba North	4	314	1	0.32
	Orumba South	1	105	0	0
	Oyi	1	105	0	0
	TOTAL	31	3167	3	0.09
Enugu	Enugu East	1	96	1	1.04
	Enugu North	1	90	0	0
	Enugu South	1	93	1	1.08
	Ezeagu	6	532	0	0
	Igbo Etiti	4	403	0	0
	Igbo Eze North	1	72	0	0
	Igbo Eze South	1	80	0	0
	Isi Uzo	2	173	0	0
	Nkanu East	1	103	0	0
	Nkanu West	4	376	0	0

State	LGA	Villages/sites (n)	Dried blood spot samples (n)	Ov16 ELISA positive (n)	Ov16 ELISA positive (%)
Enugu	Nsukka	2	155	0	0
	Oji River	3	259	0	0
	Udenu	2	186	0	0
	Udi	3	285	0	0
	Uzo Uwani	3	277	0	0
	TOTAL	35	3180	2	0.06
Imo	Aboh Mbaise	2	160	0	0
	Ahiazu Mbaise	2	145	0	0
	Ehime Mbano	2	160	0	0
	Ezinihitte	3	240	1	0.42
	Ideato North	1	75	0	0
	Ideato South	3	246	0	0
	Ihite/Uboma	2	161	0	0
	Ikeduru	2	160	0	0
	Isiala Mbano	1	81	0	0
	Mbaitoli	2	160	0	0
	Ngor Okpala	4	321	0	0
	Obowo	2	166	0	0
	Oguta	3	246	0	0
	Ohaji/Egbema	2	162	0	0
	Okigwe	4	324	0	0
	Orsu	1	78	0	0
	Oru East	1	72	0	0
	Owerri West	2	160	0	0
	Unuimo	1	80	0	0
	TOTAL	40	3197	1	0.03

Abbreviations: ELISA = Enzyme-linked immunosorbent assay; LGA = local government area

*LGAs in Nigeria are the substate administrative unit, equivalent to a district.

Table S2. Results of onchocerciasis entomological assessments in Abia, Anambra, Enugu, and Imo states, Nigeria, by local government area (LGA), 2021-2022

State	Local Government Area*	Fly collection sites Total n (productive n)**	Blackflies samples (n)	O-150 PCR positive (n)	O-150 PCR positive (%)
Abia	Arochukwu	5 (3)	6,214	0	0
	Bende	3 (1)	8	0	0
	Ikwuano	2 (1)	5	0	0
	Isiala Ngwa North	2 (0)	0	--	--
	Isiala Ngwa South	3 (0)	0	--	--
	Isiukwuato	8 (6)	1,513	0	0
	Obi Ngwa	1 (0)	0	--	--
	Ohafia	3 (0)	0	--	--
	Osisioma	2 (0)	0	--	--
	Ugwunabo	3 (0)	0	--	--
	Ukwa East	3 (0)	0	--	--
	Ukwa West	3 (0)	0	--	--
	Umu Nneochi	4 (2)	182	0	0
	Umuahia North	6 (4)	923	0	0
	Umuahia South	3 (0)	0	--	--
	TOTAL	51 (17)	8,845	0	0
Anambra	Aguata	1 (0)	0	--	--
	Anambra East	2 (0)	0	--	--
	Anambra West	3 (0)	0	--	--
	Anaocha	2 (0)	0	--	--
	Awka North	7 (4)	2,646	0	0
	Awka South	2 (0)	0	--	--
	Ayamelum	7 (4)	8,107	0	0
	Dunukofia	1 (0)	0	--	--
	Ekwusigo	1 (0)	0	--	--
	Idemili North	1 (0)	0	--	--
	Idemili South	1 (0)	0	--	--
	Ihiala	6 (0)	0	--	--
	Njikoka	2 (0)	0	--	--
	Nnewi North	1 (0)	0	--	--
	Nnewi South	6 (2)	102	0	0
	Ogbaru	2 (0)	0	--	--
	Onitsha North	1 (1)	108	0	0
	Onitsha South	1 (0)	0	--	--
	Orumba North	6 (2)	189	0	0
	Orumba South	4 (3)	192	0	0
	Oyi	1 (0)	0	--	--
	TOTAL	58 (16)	11,344	0	0
Enugu	Awgu	1 (0)	0	--	--
	Enugu East	1 (0)	0	--	--
	Enugu North	1 (0)	0	--	--
	Enugu South	1 (0)	0	--	--
	Ezeagu	6 (3)	12,653	0	0
	Igbo Etiti	5 (2)	1,113	0	0
	Igbo Eze North	1 (0)	0	--	--
	Igbo Eze South	1 (0)	0	--	--

State	Local Government Area*	Fly collection sites Total n (productive n)**	Blackflies samples (n)	O-150 PCR positive (n)	O-150 PCR positive (%)
	Isi Uzo	2 (0)	0	--	--
	Nkanu East	2 (1)	105	0	0
	Nkanu West	4 (0)	0	--	--
	Nsukka	2 (1)	14	0	0
	Oji River	5 (4)	1,422	0	0
	Udenu	2 (0)	0	--	--
	Udi	3 (1)	310	0	0
	Uzo Uwani	3 (2)	907	0	0
	TOTAL	40 (14)	16,524	0	0
Imo	Aboh Mbaise	3 (0)	0	--	--
	Ahiazu Mbaise	2 (0)	0	--	--
	Ehime Mbano	2 (1)	23	0	0
	Ezinihitte	3 (0)	0	--	--
	Ideato North	4 (2)	30	0	0
	Ideato South	3 (1)	76	0	0
	Ihite/Uboma	3 (2)	146	0	0
	Ikeduru	3 (0)	0	--	--
	Isiala Mbano	1 (0)	0	--	--
	Isu	1 (0)	0	--	--
	Mbaitoli	2 (0)	0	--	--
	Ngor Okpala	5 (0)	0	--	--
	Obowo	3 (0)	0	--	--
	Oguta	4 (0)	0	--	--
	Ohaji/Egbema	4 (0)	0	--	--
	Okigwe	12 (12)	15,121	0	0
	Orsu	1 (0)	0	--	--
	Oru East	1 (0)	0	--	--
	Owerri West	2 (0)	0	--	--
	Unuimo	2 (1)	78	0	0
	TOTAL	60 (19)	15,474	0	0

Abbreviations: PCR = polymerase chain reaction

*Local government areas are the substate administrative unit in Nigeria, similar to a district
**213 sites were prepared for fly catching. Only 66 sites (31%) yielded any flies and are counted as 'productive' sites.
